# The impact of Timothy's Law on hospitalization among patients with mental health conditions in New York State

**DOI:** 10.1186/s13033-022-00535-w

**Published:** 2022-05-21

**Authors:** Mingfei Li, Victor S. Y. Lo, Piaomu Liu, Eric Smith

**Affiliations:** 1 Department of Mathematical Sciences, Bentley University, Waltham, MA USA; 2 Workplace Investing, Fidelity Investments, Boston, MA USA; 3 Center for Healthcare Organization and Implementation Research (CHOIR), Bedford VA Healthcare System, Bedford, MA USA; 4 Departments of Psychiatry and Population and Quantitative Health Sciences, University of Massachusetts Medical School, Worcester, MA USA

**Keywords:** Mental health services, Mental care disparities, Policy evaluation, Causal inference

## Abstract

**Background:**

Timothy's law to reduce mental health care disparities was enacted in January 2007 in New York state (NY). According to Timothy's law, "if a patient is suffering from a Biologically Based Mental Illness, or is a Child with Serious Emotional Disturbances, the Inpatient mental health benefit will be the same as for any other illness". An assessment of its impact on inpatient mental health care is lacking. We provide a rigorous study of this policy intervention’s effect over the first year of its implementation.

**Methods:**

We used a quasi-experimental design to combine the difference-in-difference method and propensity score weighting. Data are from inpatient records in NY and California (CA) (as a control) between January 2006 to December 2006 (the pre-enactment year in NY) and January to December 2007 (the enactment year) for non-Medicare/Medicaid patients hospitalized in both years with specific illnesses covered by Timothy's Law. Change in length of stay from 2006 to 2007 was measured for each patient, and the differences observed in NY and California were compared to each other (Difference-in-Difference), with differences in the characteristics of patients in NY and California addressed through Propensity Score Weighting (PSW).

**Results:**

Before Timothy's Law was enacted (2006), length of stay (LOS) in NY was 16.3 days on average, and length of stay per hospitalization (LOSPH) was 11.72 days on average for the 1237 patients under study in 2006. In 2007, LOS increased by 4.91 days in NY (95% CI (2.89, 7.01)) compared with similar patients in California, and LOSPH by 3.25 days (95% CI (1.96, 4.57)). Among patients with serious mental illness diagnoses, LOS in NY increased by 7.07 days (95% CI (4.15, 10.17)), and LOSPH by 4.04 days (95% CI (1.93, 6.03)) compared to California.

**Conclusions:**

Our study strongly suggests that, within the time frame of just a single year, Timothy's Law significantly increased inpatient mental healthcare utilization in NY. Our study raises the possibility that similar laws in other locations could have similar effects.

**Supplementary Information:**

The online version contains supplementary material available at 10.1186/s13033-022-00535-w.

## Background

With the current COVID-19 pandemic, health care parity has become an increasing public concern in the media, despite the previously reported improvement of parity through health care coverages due to the Affordable Care Act (ACA) [7].^1^ "narrowing health disparities is key to improving our nation's overall health and reducing unnecessary health care costs…" was suggested by [3]. For example, in terms of mental health care disparity, a rural–urban gap has been revealed during the COVID19 pandemic and called for more needs to fill this gap in [34]. The impact of the pandemic on mental health care access and the disparity of care access among patients' races was reported in [19]. Reducing mental care disparity needs effective policies, which benefits from a rigorous evaluation of past policies addressing the same issue. Existing studies have focused on the care of children on health status, and on racial or gender groups, such as [1, 13, 21, 26, 33]. One approach to reducing mental health care disparities is to require better health coverage (increase benefits for care and intervention) of mental health conditions from insurance providers [5, 17]. According to Healthcare.gov, ‘health coverage’ refers to “Legal entitlement to payment or reimbursement for your health care costs, generally under a contract with a health insurance company”. This coverage issue is one of the important factors of mental care disparity.

“Timothy’s Law”, which was launched in the state of New York (NY) starting from January 1, 2007, then became permanent in 2009, is intended to reduce mental care disparities in the state. The law was passed in 2006 in response to the death of a NY boy Timothy O'Clair who committed suicide at the age of 12. A potential contributor to the tragedy was that the insurance plan of Timothy’s parents failed to provide sufficient coverage to fully address his mental health symptoms.

Timothy’s Law ensures the provision of mental health benefits by large group health plans that provide surgical and medical benefits in NY. These plans may not have these coverages since the federal MHPA (The Mental Health Parity Act) in 1996 does not require large group health plans to offer mental health benefits. This means that individuals who suffer from certain mental health illnesses identified in the statute, such as bipolar disorder, schizophrenia, and severe depression were mandated to receive full parity of health benefits. Patients are now covered with the same limits concerning day and visit benefits, cost-sharing, and other coverage terms that apply under their individual contracts for physical illness and injury. Full parity in large group health plans also included health coverage of children with severe emotional disturbances. For small group health plans, the law requires minimal health coverage for the length of stay of inpatient services and the number of visits of outpatient services. For children with severe emotional disturbances, small group programs were given opportunities to purchase additional health coverage at extra costs, allowing for full parity, as well [10].

Although the law’s intention was clearly to reduce disparities in care for mental health [6], the impact of Timothy’s Law has not been thoroughly examined. The NY State Insurance Department [8] published a study that focused on the cost and effectiveness of the law and found that the law expanded the health coverage of mental health benefits. The study concluded NY’s mental health parity depended largely on Timothy’s Law [8]. However, an assessment that evaluates the cause-and-effects of the policy on access to care and utilization of mental health services has not been conducted. Understanding the potential causal relationship between Timothy’s law and the usage of mental health services is an important question for policymakers and public health researchers.

Our study is a statistical analysis to explore the relationship of Timothy’s law on access to care and utilization of mental health services using a causal inference approach: the difference-in-difference method combined with propensity score weighting. Our study contributes to the current evaluation of public policies by demonstrating a statistical framework to assess and quantify causal inferences about the relationships of a policy on its targeted outcomes. In our analysis, we studied the inpatient population who were hospitalized due to the mental health illnesses statutorily identified by Timothy’s Law in the state of New York in 2007. Like other published policy studies [23, 24, 29, 30], we used Length of Stay as the measure of service utilization during hospitalization. We examined the effects of the enactment of Timothy’s Law starting January 1, 2007, on the outcomes of the total length of stay (LOS) and average length of stay per hospitalization (LOSPH) for each patient in NY compared to a control group of inpatients in California (CA), who experienced no pronounced changes in mental health policy.

## Methods

### Study population

We collected all patient-level data with corresponding mental health diseases addressed by Timothy’s Law from State Inpatient Databases (SID), Healthcare Cost and Utilization Project (HCUP) [14], Agency for Healthcare Research and Quality in both 2006 and 2007 for New York State and California State, based on International Classification of Diseases, Ninth Revision (ICD-9) codes: Schizophrenia (including schizoaffective) disorders (ICD-9 code 295.x), Major depression (ICD-9 code 296.2,296.3, 311.x), Bipolar disorder ((ICD-9 code 296.4, 296.5, 296.6, 296.7, 296.89), Delusional disorders (ICD-9 code 297.1), Panic disorder (300.01), Obsessive–compulsive disorder (300.3), Bulimia (307.51), Anorexia (307.1) (Additional file 1: Table S1). Due to the sample size limitation, we decided to use one year before and after the policy intervention as the pre-period and post-period to examine the effect of this intervention, i.e., 2006 and 2007. Also, since the policy under this study targets private insurance, we focus on patients who used private insurance as their primary payer during hospitalization. After excluding patients who only have mental health hospitalizations in either 2006 or 2007 and patients who did not use any private insurance as the primary payer, such as Medicare, Medicaid, self-pay and others, we have N = 1237 patients with mental health disorders from NY and N = 3028 from CA (California) who had at least one inpatient hospitalization for mental health conditions in both 2006 and 2007. As a secondary analysis, we also studied the subcohort of patients who suffered from serious mental illness [schizophrenia (295.x), schizoaffective disorder (295.7), and bipolar disorder (296.4, 296.5, 296.6, 296.7 and 296.89)] with N = 620 in NY and N = 1,628 in CA.

### Hospitalization

As a proxy for the utilization of the health care services in hospitalization, we use total length of stay (LOS) per patient within the calendar year and length of stay per hospitalization (LOSPH) per patient as the outcome measures in this study. The differences in the outcome between 2006 (pre policy implementation) and 2007 (post policy implementation) were computed and compared between NY (intervention) and CA (control).

### Demographics and comorbidities

In all our models, we applied adjustment of demographics (age, sex, race) and baseline comorbidities to control for potential confounding errors. These comorbidities include Asthma, Sleep, Thyroid, Obesity, Tobacco, TBI (Traumatic Brain Injury), Cardiac Dysrhythmia, Cancer, Congestive Heart Failure, Coronary Artery Disease, Diabetes, Hyperlipidemia, Hypertension, Kidney, Liver, Lung, Peripheral Artery Disease, Stroke, Alcohol Dependency, Anxiety, Dementia, PTSD (Post-traumatic stress disorder) and Substance Dependency.

### Quasi-experimental study design

While randomized controlled trials are the gold standard for causal inference, techniques have been developed for drawing causal inferences when randomized trials are not possible or were not done. One powerful causal inference technique is the Difference-in-Difference (DID) method. DID simply starts with a pre/post comparison in the intervention group to examine outcomes before and after the intervention is available. A pre-post comparison allows even unmeasured confounding factors which do not change across the time period examined to be controlled since each patient is compared with themselves (e.g., their genetics, family, and past history before the time periods that are compared will be identical). To control for large-scale secular trends (e.g., a tendency for length of stay in the United States to shorten over time), a similar pre/post difference is calculated for a control location. The control location in this study was California, a state that had already passed a law similar to Timothy’s law years earlier. Although California had a similar policy already before the study period, it didn’t have a similar policy intervention at the same period. Thus, we use it as a control group in this study and used a patient-level DID approach to estimate the effect of the policy’s intervention in New York. We include a more detailed mathematical explanation in the Additional file 1: Appendix.

The DID calculation involves the difference between those two differences seen within each sample, see [2, 16], and [11]. While the DID can control for some unmeasured confounding, which is a powerful attribute, it does depend on the assumption that without the intervention, utilization would change to the same amount as it did for the control group (CA patients). The control group does not necessarily need to be similar to the intervention group, as long as the “parallel trend” assumption is met, although this can never be known with certainty. To improve the likelihood of meeting the “parallel trend” assumption, we integrated DID with another approach often used to improve causal inferences in nonrandomized studies, Propensity Score Weighting (PSW). Propensity Score Weighting can balance samples across a large number of measured covariates [15, 18, 20, 22, 27]. Although PSW and its variants are widely popular in social sciences and medical sciences due to its practicality, to be fully valid, PSW does require the assumption that all confounders are observable and available (which also cannot be known with certainty). By combining DID and PSW, both approaches can help complement each other and improve the validity of nonrandomized comparisons even if assumptions are not perfectly met. This approach of combining PSW and DID methods has been used to evaluate the impacts of health insurance payment innovations [32], sleep quality [9], the impact of Medicare & Medicaid policies on diabetes readmission rates [25], health care utilization and costs associated with Traumatic Brain Injuries among US Veterans [31], changes in service utilization for youths [12], home visit for newborns [35], and diabetes medication adherence [37]. In our study, we apply a similar approach to study the effect of Timothy’s Law’s impact on inpatient health service utilization.

### Statistical analysis

Using the general method described above, we examined the outcomes of LOS and LOSPH. First, we extracted the mental health patients who had a mental health hospitalization record and chose private insurance as the primary payer in both 2006 and 2007 in NY or CA. Then, we computed the total days of hospitalization for each patient to be his/her LOS, and used this LOS divided by the total number of hospitalizations in each calendar year to be this patient’s LOSPH. Then, for each patient, we computed the LOS difference and LOSH difference between 2006 and 2007.

In order to improve our comparison between the state that received the intervention in 2007 (NY) and the state that did not (CA), we sought to balance the patient characteristics of intervention (NY) and control groups (CA) and thus improve control for potential confounders. We used both patients’ demographic information (age, sex, race) and selected comorbidities at baseline in the PSW model to compute the propensity scores, which were used to adjust the final DID model to estimate the effect of Timothy’s Law. This approach minimizes the chance of mis-estimating the intervention effect due to differences in confounders between the two patient samples. SAS PROC CAUSALTRT is used to estimate the Average Intervention Effect on the Treated (ATT) using inverse probability weighting (IPW). In our case, this effect (ATT) is the average intervention effect of this policy in the state of New York, i.e., the average difference in outcome between having Timothy’s Law and not having the law in New York.

## Results

New York State had 1,237 patients hospitalized in both 2006 and 2007 because of their mental health problems listed by Timothy’s Law, while 3,028 patients met this criterion in California (Table 1). Comparing these two states, the patient sample in California was slightly younger, had a higher prevalence of males, and more patients self-identifying as having Hispanic ethnicity and fewer patients self-identifying as African American. Table 1 also shows that after PSW, differences in all the patient characteristics were decreased between the NY and CA samples, based on the commonly used standardized mean difference (SMD) measure [38].

**Table 1:** Descriptive characteristic of the two samples at baseline before and after PSW

	Before PSW					After PSW				
	NY		CA			NY		CA		
	2006, N = 1237		2006, N = 3028			2006, N = 1211		2006, N = 2345		
Variables	Mean	STD	Mean	STD	SMD	Mean	STD	Mean	STD	SMD
Age	39.16	14.93	37.68	14.35	0.101	38.37	14.75	38.42	14.06	−0.003
Length of stay	16.73	18.73	14.12	15.96	0.150	16.06	17.98	14.71	16.74	0.078
Length of stay per hospitalization	11.72	13.17	8.52	8.27	0.291	11.19	12.24	8.63	8.49	0.243
	count	%	count	%		count	%	count	%	
Race or ethnicity										
White	966	79.8%	1838	78.4%	0.034	963	79.38%	1838	78.38%	0.025
Black	147	12.1%	148	6.3%	0.202	98	8.08%	148	6.31%	0.069
Hispanic	45	3.7%	269	11.5%	−0.298	105	8.67%	269	11.47%	−0.093
Asian or pacific Islander	21	1.7%	56	2.4%	−0.049	25	2.07%	56	2.39%	-0.022
Native American	4	0.3%	2	0.1%	0.045	2	0.18%	2	0.09%	0.025
Other	28	2.31%	32	1.36%	0.071	20	1.63%	32	1.36%	0.022
Female	760	61.4%	1682	55.5%	0.120	776	64.1%	1484	63.3%	0.017
Bipolar	448	36.2%	1133	37.4%	−0.025	436	36.0%	882	37.6%	−0.033
Schizophrenia	263	21.3%	616	20.3%	0.025	231	19.1%	457	19.5%	−0.010
Depression	513	41.5%	1255	41.4%	0.002	515	42.5%	992	42.3%	0.004
Asthma	43	3.5%	154	5.1%	−0.079	67	5.51%	121	5.17%	0.015
Sleep	3	0.2%	45	1.5%	−0.142	19	1.57%	24	1.02%	0.049
Thyroid	33	2.7%	106	3.5%	−0.046	39	3.20%	81	3.46%	−0.014
Obesity	28	2.3%	130	4.3%	−0.112	48	4.00%	92	3.94%	0.003
Tobacco	36	2.9%	182	6.0%	−0.151	68	5.60%	132	5.61%	0.000
TBI	1	0.1%	2	0.1%	0.000	1	0.11%	2	0.09%	0.006
Cardiac dysrhythmia	14	1.1%	15	0.5%	0.067	9	0.75%	18	0.75%	0.000
Cancer	3	0.2%	4	0.1%	0.026	1	0.11%	2	0.09%	0.006
Congestive heart failure	4	0.3%	4	0.1%	0.045	3	0.23%	6	0.25%	−0.004
Coronary artery disease	15	1.2%	13	0.4%	0.090	9	0.76%	19	0.81%	−0.006
Diabetes	61	4.9%	99	3.3%	0.081	47	3.86%	94	3.99%	−0.007
Hyperlipidemia	38	3.1%	112	3.7%	−0.033	43	3.51%	86	3.66%	−0.008
Hypertension	82	6.6%	249	8.2%	−0.061	99	8.21%	190	8.09%	0.004
Kidney	4	0.3%	4	0.1%	0.045	3	0.25%	6	0.24%	0.002
Liver	2	0.2%	5	0.2%	0.000	3	0.28%	5	0.21%	0.014
Lung	60	4.9%	186	6.1%	−0.053	80	6.60%	150	6.38%	0.009
Peripheral artery disease	1	0.1%	1	0.0%	0.045	1	0.06%	1	0.06%	0.000
Stroke	1	0.1%	3	0.1%	0.000	1	0.08%	3	0.11%	−0.010
Alcohol dependency	113	9.1%	392	12.9%	−0.122	144	11.88%	292	12.45%	−0.017
Anxiety	74	6.0%	202	6.7%	−0.029	80	6.64%	161	6.86%	−0.009
Dementia	1	0.1%	2	0.1%	0.000	1	0.06%	2	0.08%	−0.008
PTSD	35	2.8%	112	3.7%	−0.051	40	3.31%	89	3.78%	−0.025
Substance dependency	109	8.8%	314	10.4%	−0.054	126	10.37%	239	10.18%	0.006

In Table 2, we compare the difference of total hospitalization time for each patient’s hospital stays in 2006 versus 2007. The NY patients’ total hospital stays for mental illness averaged 5.14 days longer in 2007 than in 2006 (95% CI (3.52, 6.76), p-value < 0.0001) after Timothy’s law was implemented (Table 2). The California patients’ hospital stays increased on average only 0.73 days in 2007 compared to 2006 (95% CI (0.021, 1.44), p-value = 0.044). We also estimated the LOSPH (total Length of Stay divided by the number of hospitalizations) and reported the difference between 2006 and 2007 in Table 2. The NY patients received 4.01 (95% CI (2.85, 5.16), p-value < 0.0001) more days per hospitalization compared with 2006, while CA patients’ care was 0.79 days (95% CI (0.36, 1.22), p-value = 0.0003) longer per hospitalization compared with the previous year (Table 2). Even greater changes were observed in LOS and LOSPH in our secondary analysis of patients with Severe Mental Illness (Table 2).

**Table 2:** Difference in Length of stay (LOS) and Length of stay per hospitalization (LOSPH) by State, 2007 compared to 2006 for Mental health illnesses covered by Timothy’s Law, prior to propensity-score weighting

All mental health illnesses covered by Timothy’s Law								
	New York, N = 1237, LOS of 2006 = 16.73 LOSPH of 2006 = 11.72				California, N = 3028, LOS of 2006 = 14.12 LOSPH of 2006 = 18.52			
Variable	Mean	Std Dev	95% CI of mean	p-value	Mean	Std Dev	95% CI of mean	p-value
diff_LOS	5.14	29.06	(3.52,6.76)	< 0.0001	0.73	19.93	(0.02,1.44)	0.044
diff_LOSPH	4.01	20.69	(2.85,5.16)	< 0.0001	0.79	12.00	(0.36,1.22)	0.0003

Severe mental illnesses								
	New York, N = 620 LOS of 2006 = 18.82 LOSPH of 2006 = 13.70				California, N = 1628, LOS of 2006 = 15.92 LOSPH of 2006 = 9.79			
Variable	Mean	Std Dev	95% CI of mean	p-value	Mean	Std Dev	95% CI of mean	p-value
diff_LOS	6.52	32.48	(3.95,9.08)	< 0.0001	0.74	21.81	(−.32,1.80)	0.1735
diff_LOSPH	4.91	23.75	(3.04,6.78)	< 0.0001	1.05	14.23	(0.36,1.75)	0.0028

diff_LOS = LOS of 2007-LOS of 2006; diff_LOSPH = LOSPH of 2007-LOSPH of 2006

Using PSW to balance our two samples on both demographic information and patients’ comorbidities prior to calculating the Difference-in-Difference, we were able to estimate the effect of Timothy’s Law (Table 3) for NY compared with the control group (CA) on both total length of stay (LOS) and the LOSPH. The estimated effect is 4.91 more days in total LOS with 95% CI (2.89, 7.01) from the bootstrap method and p-value < 0.0001, which is statistically significantly higher than that in the control group (CA). Similarly, the estimated effect of Timothy’s Law on LOSPH is 3.25 more days per hospitalization compared with the control group, with 95% CI (1.94, 4.57) and p-value < 0.0001. This indicates the significant impact of Timothy’s law on providing more inpatient care on mental health patients who were covered by private insurance. The result for patients with severe mental health illness is consistent with the above. The estimated effect on total LOS was 7.07 days with 95% CI (4.15, 10.17) and p-value < 0.0001. For the LOSPH, this estimated effect was 4.04 days with 95% (1.93, 6.03) and p-value = 0.0002. Both effects were statistically significant compared with the control group. All 95% CIs were obtained from the bootstrapping process.

**Table 3:** Policy intervention effect on length of stay and length of stay per hospitalization (PS weighted difference-in-difference)

All mental health illnesses covered by Timothy’s Law								
Outcome variable	Total length of Stay (LOS)				Length of stay per hospitalization (LOSPH)			
Parameter	Estimate	Bootstrap 95% CI		p-value	Estimate	Bootstrap 95% CI		P-value
Intervention	5.23	3.64	6.86	< .0001	3.99	2.87	5.2	< .0001
Control	0.31	−0.81	1.58	0.58	0.74	0.13	1.37	0.0146
Policy's effect (ATT)	4.91	2.89	7.01	< .0001	3.25	1.96	4.57	< .0001

Severe mental health illnesses								
Parameter	Estimate	Bootstrap 95% CI		p-value	Estimate	Bootstrap 95% CI		P-value
Intervention	6.63	4.17	9.32	< .0001	3.14	6.79	3.14	< .0001
Control	−0.44	−2.09	1.04	0.5678	−0.47	1.83	−0.47	0.0746
Policy's effect (ATT)	7.07	4.15	10.17	< .0001	1.93	6.03	1.93	0.0002

The results were adjusted by age, race, sex, and all comorbidities

In this study, we integrated Propensity Score Weighting (PSW) and Difference-in-Difference (DID) approaches to study the policy intervention, and the latter assumes that the intervention and control groups would have followed the same trend (e.g., increasing or decreasing) if the intervention group did not receive the intervention. When something other than the intervention changes in one group but not the other, it will be a violation of the DID assumption. This is an assumption that cannot be formally tested. Nevertheless, we examined the monthly rates of change in length of stay over the year 2006 in both states, and between 2006 and 2007 in California, and the evidence suggested that the two states have similar changes in 2006. The trends of CA and NY in 2006 have no significant difference with a p-value = 0.42. Also, the slopes of CA in 2006 and 2007 have no significant difference with a p-value = 0.89 (Table 2). At a minimum, the DID changes we observed were driven almost entirely by large changes in NY’s LOS and LOSPH, rather than from changes occurring in CA (in which LOS and LOSPH were relatively consistent between 2006 and 2007) (Additional file 1: Table S2).

## Discussion

In this study, we used the patient-level data from the HCUP State inpatient databases for NY and CA to evaluate the potential causal relationship of Timothy’s Law and mental health patients in NY, who are the target beneficiaries of this policy. To estimate this effect, we employed an integrated, individual-level pre-post design, Propensity Score Weighting (PSW), and the Difference-in-Difference (DID) method on mental health patients’ hospitalization in 2006 and 2007. Through our analysis, we identified a significant, potentially causal relationship of Timothy’s Law and the outcome measure(s) of length of stay for inpatient mental health care. This study also provides an example of how causal inference can be employed in policy evaluation. The statistical tools under the causal-inference paradigm in our work can be applied to a broad range of policy evaluations, especially those involving policies with discrete implementation dates.

The inpatient length of stay has been used as an outcome measure for previous health policy studies. For example, [28] studied multiple inpatient outcomes, including length of stay for emergency department utilization. [30] found that the LOS in the emergency room was influenced by patient’s self-pay status, which is related to insurance coverage. Similarly, [24] also found that patients with mental health conditions in Massachusetts had LOS that varied by insurance coverages. [29] studied the length of stay in heart failure in US and Canada after the Hospital Readmissions Reduction Policy implemented from 2010. They found no association between the change of length of stay in heart failure and this policy. [23] also examined the factors associated with length of stay in mental health services in London, UK, through multiple regressions and found demographic factors, such as gender and race, and different mental health diagnoses have a significant association with length of stay.

However, there are no published studies evaluating the impact of Timothy’s Law policy intervention on patient outcomes, such as on inpatient hospitalization in New York state. We sought to advance the rigor of studies of mental health policy by applying three complementary methods (an individual-level pre-post, difference-in-difference design and propensity score weighting) to increase the likelihood of minimizing the impact of confounding and making valid causal inferences about the effect of Timothy’s Law.

For the outcome measures of our study, we adjusted patients’ demographic information and comorbidities at baseline and found that Timothy’s Law significantly increased the inpatient care utilization of mental health patients in terms of both LOS and LOSPH. The changes we observed after the enactment of Timothy’s Law (compared with the control group), which involved more than a 25% increase in overall length of stay across the year and of length of stay per hospitalization, are both statistically and clinically significant.

Although the intention of the Mental Health Parity Act of 1996 and the Mental Health Parity and Addiction Equity Act of 2008 (MHPAEA), and the ACA are to significantly improve the health coverage of and access to mental health services, it is not clear how thoroughly this goal has been achieved, [4]. As just one example, our study demonstrated that a law (Timothy’s Law) enacted after the MHPA (The Mental Health Parity Act) of 1996 still was able to have a dramatic impact on health service use, implying the 1996 MHPA on its own did not resolve disparities in NY state. It is possible that further study of the ways in which Timothy’s Law complemented or extended these federal laws to enhance inpatient mental healthcare utilization may help reveal approaches to further improve mental healthcare parity and utilization.

Our study, just like any nonrandomized study of an intervention, has a number of limitations. Since we were limited to nonrandomized (i.e., observational) data concerning the impact of Timothy’s Law, insufficient control of confounding is always possible. However, a strength of our study is that we applied a combination of causal inference techniques in an effort to limit any residual confounding to a minimum. First, our pre-post design removes the effects of fixed confounders (e.g., a patients’ race, genetics, childhood and developmental history, family history, and) on our estimates. Second, because we chose a narrow time window for our comparison (one year compared to the immediately preceding year), even time-varying confounders that did not vary within the period of those 2 years would be expected to be partly or fully controlled (e.g., mental illness severity up until 2006, physical illness severity up until 2006, etc.).

Third, to control for secular trends (a factor external to an individual patient’s characteristics) that could bias our results, such as an overall trend to shorten or lengthen inpatient length of stay, we adopted a Difference-in-Difference design. As pointed out above, ongoing implementation of initiatives such as the MHPA in 1996, or general economic factors, may influence measures such as length of stay. The effectiveness of this method to address secular trends depends on the degree to which the comparison state, California, has a parallel trend to New York in length of stay. This can never be determined with certainty since it is impossible to observe what the trend would have been in New York State without the intervention, but we did perform some suggestive analyses that indicated the trend in length of stay month-to-month over the 2006 year was in the same direction in New York state and California and not significantly different (p = 0.42). Furthermore, the change we observed in 2007 in California in Length of Stay was > 6% of the change seen in New York state, suggesting that secular trends were modest and that the vast majority of the change in length of stay observed in New York state in 2007 was due to the effect of Timothy’s Law. We also chose California not only because it had no large-scale policy changes, to our knowledge, concerning mental health patients during our study, but it also had passed a law similar to Timothy’s Law years prior, meaning that local initiatives (e.g., at the hospital level) were less likely to produce large changes in length of stay. It is possible, however, that use of a different comparison group than California patients, or use of multiple comparison groups, would have further strengthened our study.

Fourth, to address the possibility of secular changes within particular subsets of our sample, we used propensity score weighting to balance the New York state and California patients. The PSW weighting also helps prevent the estimated changes in length of stay from differing due to different patient characteristics between the two samples by balancing those characteristics across the two samples. Our PSW worked very well, achieving 68% reduction, on average in the standardized mean differences observed in patient characteristics between New York and California prior to PSW. (For example, patients with Hispanic ethnicity in California were over 3 times of those in the New York state prior to PSW, but after PSW, this difference was reduced so that the California sample had only 32% higher than that of New York in terms of Hispanic %). However, no method can be expected to completely remove differences in patient characteristics in all circumstances, debates exist on whether Propensity score weighting or other propensity score methods more reliably minimize confounding, and the PSW technique is not able to balance the two samples on unmeasured factors, however.

Other study limitations relate to external validity (generalizability) and data limitations. First, because we used a patient-specific pre-post difference in length of stay as our outcome measure, we only evaluate the effect of Timothy’s Law on patients who were hospitalized both in 2006 and 2007. We are unable to make inferences concerning its impact on patients who were just hospitalized in 2007, the year the Law took effect, but not 2006. Second, we only examined patients who were privately insured; thus, our results may not generalize to patients with other insurance. Furthermore, we classified patients as privately insured based on the primary payer listed in their inpatient records. This designation may have been in error sometimes, leading to some degree of misclassification of our study sample. Third, due to the limitation of data, we were not able to adjust for the patient’s living status, such as living alone. Similarly, due to the sample size limitation, we were able to use only one year period as the post period after the policy intervention. Finally, we were unable to examine the impact of Timothy’s Law on outpatient care, due to the limitation of our data.

## Conclusion

In summary, using multiple causal inference approaches, our study provides strong evidence that a state-level initiative, Timothy’s Law, did significantly increase the inpatient care utilization of the mental health patients in New York state who were covered by private insurance. Our study also suggests that the potential causal effect of this intervention and other similar health policy interventions can be meaningfully evaluated through causal inferences approaches such as those we have applied in this study.

## Supplementary Information

**Additional file 1: Table S1.** ICD-9 code list for all diseases **Table S2** Trends of monthly LOS in NY in 2006, and CA in 2006 and 2007

## Data Availability

The data used in this study is not publicly available since any data usage or access needs HCUP approval. Data are available from the authors upon reasonable request and with the permission of HCUP.

## Abbreviations

US: The United States
UK: The United Kingdom
NY: New York State of United States
CA: California State of United States
PSW: Propensity score weighting
LOS: Length of Stay
LOSPH: Length of stay per hospitalization
CI: Confidence interval
ACA: The affordable care act, the comprehensive health care reform law enacted in March 2010 (sometimes known as ACA, PPACA, or “Obamacare”).
MHPA: The mental health parity act of 1996
MHPAEA: The Paul wellstone and pete domenici mental health parity and addiction equity act of 2008
HCUP: Healthcare cost and utilization project
SID: State inpatient databases
ICD-9: International classification of diseases, ninth revision.
TBI: Traumatic brain injury
PTSD: Post-traumatic stress disorder
DID: Difference-in-difference method
ATT: Average intervention effect on the treated
IPW: Inverse probability weighting
SMD: Standardized mean difference

## Footnotes

1 For example, the dependent provision of ACA is associated with the reduction of emergency room visits on psychiatric issues among young adults [36].
