# Supplementary material for: The impact of Timothy's Law on hospitalization among patients with mental health conditions in New York State
Source: Int J Ment Health Syst. 2022 May 21;16:25. doi: 10.1186/s13033-022-00535-w (PMC9124051; doi:10.1186/s13033-022-00535-w)
Supplement: Supplementary file 1 — Additional file 1: Table S1. ICD-9 code list for all diseases Table S2 Trends of monthly LOS in NY in 2006, and CA in 2006 and 2007 [file 13033_2022_535_MOESM1_ESM.docx]

**Appendix:**

In a typical Difference-in-Difference (DID) design setting, the treated group received an intervention between the pre and post periods while the comparison or control group did not receive an intervention at all. In our data, however, California actually introduced the law (CMHPA) in 1999 which is similar to Timothy’s Law in New York. As a result, California is not a typical control group. Under the assumption that the effect due to time difference is the same in both states (i.e., the common trend), the DID methodology can still be justified as follows.

Define:

$Y_{NY,1}\left( T \right)=$ post Timothy’s Law period outcome in New York (*T* denotes treated),

$Y_{NY,0}\left( C \right)=$ pre Timothy’s Law period outcome in New York (*C* denotes untreated),

$Y_{CA,1}\left( T \right)=$ post Timothy’s Law period outcome in California (*T* denotes treated),

$Y_{CA,0}\left( T \right)=$ pre Timothy’s Law period outcome in California (*T* denotes treated because California had a similar policy in place before Timothy Law became effective in New York),

$\lambda_{t}=$ mean change in outcome due to only time difference, assumed to be the same for California and New York, i.e., a common trend,

δ = mean change in outcome due to policy, i.e., the average intervention effect that we are interested in estimating.

Then,

$$Y_{CA,1}\left( T \right)=\lambda_{t}+Y_{CA,0}\left( T \right)+ \varepsilon_{CA}, \left( 1 \right)$$

where $\varepsilon_{CA}$ is the random error in California in the post-Timothy Law period, and

$Y_{NY,1}\left( T \right)=\lambda_{t}+Y_{NY,0}\left( C \right)+$ δ $+ \varepsilon_{NY}$, (2)

where $\varepsilon_{NY}$ is the random error in New York in the post-Timothy Law period.

From (1), $\lambda_{t}$ = ${E(Y}_{CA,1}\left( T \right))-E(Y_{CA,0}\left( T \right))$. (3)

And from (2),

$$\delta={E(Y}_{NY,1}\left( T \right))-E\left( Y_{NY,0}\left( C \right) \right)-\lambda_{t}$$

$=({E(Y}_{NY,1}\left( T \right))-E\left( Y_{NY,0}\left( C \right)) \right)-({E(Y}_{CA,1}\left( T \right))-E(Y_{CA,0}\left( T \right)))$ , using (3),

which shows that $\delta$ is simply the DID estimator and can be estimated in the regular fashion.

**Supplementary tables and figures**

**Supplementary Table 1: ICD-9 code list for all diseases**

| **Disease** | **ICD9 code** |
| --- | --- |
| **Bipolar** | 296.00, 296.01, 296.02, 296.03, 296.04, 296.05, 296.06, 296.10, 296.11, 296.12, 296.13, 296.14, 296.15, 296.16, 296.40, 296.41, 296.42, 296.43, 296.44, 296.45, 296.46, 296.50, 296.51, 296.52, 296.53, 296.54, 296.55, 296.56, 296.60, 296.61, 296.62, 296.63, 296.64, 296.65, 296.66, 296.80, 296.81 , 296.89, 296.90, 296.99, 296.7 |
| **Schizophrenia** | 295 |
| **Depression** | 311, 296.2, 296.3, 300.4, 296.82 |
| **Asthma** | 493 |
| **Sleep disorder** | 307.42, 780.52 |
| **Thyroid** | 244 |
| **Obesity** | 278.0, 649.1 |
| **Tobacco** | 305.1, 649.0 |
| **TBI** | 310.2, 905.0,907.0, 800, 801, 803, 850, 851, 852, 853, 854, 804, 959.01 |
| **Cardiac Dysrhythmia** | 427.0, 427.1, 427.2, 427.3, 427.4, 427.8 |
| **Cancer** | 140 to 165, 170 to 172, 174 to 176, 179 to 184, 186 to 208, 209 to 209.3, 239 |
| **Congestive Heart Failure** | 398.91, 402.01, 402.11, 402.91, 404.01, 404.03, 404.11,404.13, 404.91, 404.93, 425, 428 |
| **Coronary Artery Disease** | 410 to 414, 429.2 |
| **Diabetes** | 249, 250, 648.00 |
| **Hyperlipidemia** | 272.0, 272.1, 272.2, 272.3, 272.4 |
| **Hypertension** | 401 to 405 |
| **Kidney** | 581, 582, 583, 585, 586, 587, 588.8, 588.9 |
| **Liver** | 5722, 5723, 5724, 5728, 4560, 4561, 4562, 5712, 5714, 5715, 5716, 5718, 5719,7895, V427, 567.23 |
| **Lung** | 490 to 494, 496, 500 to 505, 506.4 |
| **Peripheral Artery Disease** | 440, 441, 442,443.89, 443.9, V434 |
| **Stroke** | 436, 438, 433.01, 433.11, 433.21, 433.31, 433.81, 433.91, 434.01, 434.11, 434.91 |
| **Alcohol Dependency** | 291, 291.0, 291.1, 291.2, 291.3, 291.4, 291.5, 291.8, 291.81, 291.89, 291.9, 303, 303.0, 303.00, 303.01, 303.02, 303.03, 303.9, 303.90, 303.91, 303.92, 303.93, 305.0, 305.00, 305.01, 305.02, 305.03, 357.5, 425.5, 535.3, 535.30, 535.31, 571.0, 571.1, 571.2, 571.3, 790.3, 980, 980.0, 980.8, 980.9, E86.0, E86.00, E86.01, E86.08, E86.09, V11.3 |
| **Anxiety** | 300.00, 300.01, 300.02, 300.09, 300.10, 300.20, 300.21, 300.22, 300.23, 300.29, 300.3 |
| **Dementia** | 290.0, 290.10, 290.40, 290.41, 290.42, 290.43, 294.10, 294.11, 331.19, 331.2, 331.7, 331.82, 331.83, 331.89, 331.9, 797 |
| **PTSD** | 309.81 |
| **Substance Dependency** | 292, 304.2, 304.4, 304.5, 304.6, 304.7, 304.8, 304.9, 305.3, 305.4, 305.5, 305.6 , 305.7, 305.9 |

**Supplementary table 2 Trends of monthly LOS in NY in 2006, and CA in 2006 and 2007**

|  | **Regression slope of Monthly LOS** | **std err** | **95% CI** | | **Trends compare: CA2006 VS NY 2006 (p-value)** | **Trends compare: CA2006 VS CA 2007 (p-value)** |
| --- | --- | --- | --- | --- | --- | --- |
| **NY in 2006** | -0.033 | 0.032 | -0.10 | 0.04 | 0.42 |  |
| **CA in 2006** | -0.064 | 0.019 | -0.11 | -0.02 |  | 0.89 |
| **CA in 2007** | -0.068 | 0.022 | -0.12 | -0.02 |  |  |
